# A pilot study of a portable hand washing station for recently displaced refugees during an acute emergency in Benishangul-Gumuz Regional State, Ethiopia

**DOI:** 10.1186/s13031-015-0053-6

**Published:** 2015-08-22

**Authors:** Farah Husain, Colleen Hardy, Lemlem Zekele, David Clatworthy, Curtis Blanton, Thomas Handzel

**Affiliations:** Emergency Response and Recovery Branch, Center for Global Health, Division of Global Health Protection, Centers for Disease Control and Prevention, 1600 Clifton Road, NE, MS E-22, Atlanta, GA 30329 USA; International Rescue Committee, T.K. International P.L.C, Bole Sub-city, Kebele 03/05, House # 162, Addis Ababa, Ethiopia

**Keywords:** Hand washing, Refugees, Humanitarian emergencies, Ethiopia, WASH

## Abstract

**Background:**

Diarrheal disease is a common cause of morbidity and mortality. Displaced populations are especially vulnerable due to overcrowded camps and limited access to water and sanitation facilities, increasing the risk for outbreaks. Hand washing with soap is effective against disease transmission, and studies suggest access to a convenient hand washing station may be the key to increasing hand washing behavior. This pilot study evaluated the acceptability, durability and use of a novel hand washing bag (HWB) at the household level among Sudanese refugees immediately following an acute emergency.

**Methods:**

We distributed one HWB to every household (*n* = 874) in Adamazin Transit Center in western Ethiopia. The evaluation consisted of baseline and endline surveys, three monthly monitoring visits and focus group discussions (FGDs) over a six month period. FGD data were analyzed using the Risk, Attitudes, Norms, Abilities, and Self-Regulatory model. Survey and monitoring data were analyzed using SPSS. Note: Residents were resettled to Bambasi Refugee Camp during the study period where the endline survey was conducted.

**Results:**

Baseline data suggested water quantity and availability of soap were below SPHERE standards, however participants responded positively to the HWB. At the end of the monitoring period, 73.9 % of the same households retained their original HWBs and 66.7 % of bags had water at the time of the visit. The mean lifespan of the HWB during the monitoring period was 2.73 months. From a new sample of households selected for the endline evaluation, 93.0 % had an original HWB, but only 39.4 % had water in the bag. Endline FGD participants felt the HWB was useful, but reported insufficient soap and hygiene messaging.

**Conclusion:**

The HWB performed well during the early phases of the emergency, however longer term results in this setting are unclear. The low levels of reported use measured by proxy indicators at six months indicated decreasing acceptability over time or a reflection of potential differences between the two sites. It is also unknown whether the HWB influenced hand washing behavior. Study findings were shared with the manufacturer in an effort to improve the bag’s acceptability, utility, and durability.

## Background

Diarrhea is a leading cause of deaths among children under-five years of age worldwide, resulting in an estimated 1.2 million child deaths annually due to a lack of safe water, basic sanitation and hygiene [1]. Displaced populations are especially vulnerable to diarrheal illnesses. During an acute emergency, camps are often overcrowded and access to water and sanitation facilities is limited increasing the risk of waterborne disease transmission and potential outbreaks. Hand washing with soap is known to be one of the most effective interventions [2]. However, the United Nations High Commissioner for Refugees (UNHCR) standards for humanitarian emergency assistance recommend the provision of hand washing stations with soap next to communal latrines which addresses hand washing after latrine use. It does not mention hand washing stations at the household level to promote hand washing behavior at other critical times such as before eating or before preparing meals. It has been shown that access to a convenient hand washing station with soap is associated with higher rates of hand washing [3] and having access to water and soap at critical times may be the key to increasing hand washing behavior.

Prior to the start of this evaluation, we reviewed the Water and Sanitation Program’s (WSP) international repository on different hand washing technologies used in resource-poor settings [4, 5]. These included traditional jerry cans, buckets, and variations of the Tippy Tap. Most of these devices required some assembly and/or were not logistically feasible or easy to transport early in the emergency. We selected an inexpensive 1 simple collapsible 10 liter heavy duty plastic hand washing bag (HWB) used in households, schools, daycare facilities, and by food vendors in communities in Cape Town, South Africa. An evaluation of an earlier prototype was carried out in 2008; however, it has not been evaluated in an emergency setting [6]. A similar technology, the Bush Proof hand washing container, was piloted in a non-emergency setting in Zimbabwe, but did not have an attached soap pouch or pictorial instructions [7].

Ethiopia hosts a number of displaced populations and experienced an influx of Sudanese refugees in 2011, due to on-going civil war along their western border with Sudan. Between September 2011 and February 2013, approximately 90,000 people fled the neighboring Blue Nile State in Sudan to western Ethiopia due to fighting between the Sudanese Army and the rebels of the northern South Sudan People’s Liberation Army (SPLA-N) [8]. A new camp along with several transit centers opened in Benishangul-Gumuz Regional State (BGRS) near the Sudan border to accommodate the refugees. The Administration for Refugee and Returnee Affairs (ARRA), UNHCR and the International Rescue Committee (IRC), along with other partners provided basic water, sanitation, and hygiene (WASH) services to the newly established Adamazin Transit Center such as latrines, safe water and hygiene education. Hand washing stations at the communal or household level were not present.

We undertook this evaluation to assess the acceptability, durability and use of the HWB during the initial phases of an acute emergency when basic services were scarce. The availability of a hand washing station with soap at the household level may provide an enabling environment to increase hand washing behavior.

## Methods

### Evaluation design

**Fig. 1:**
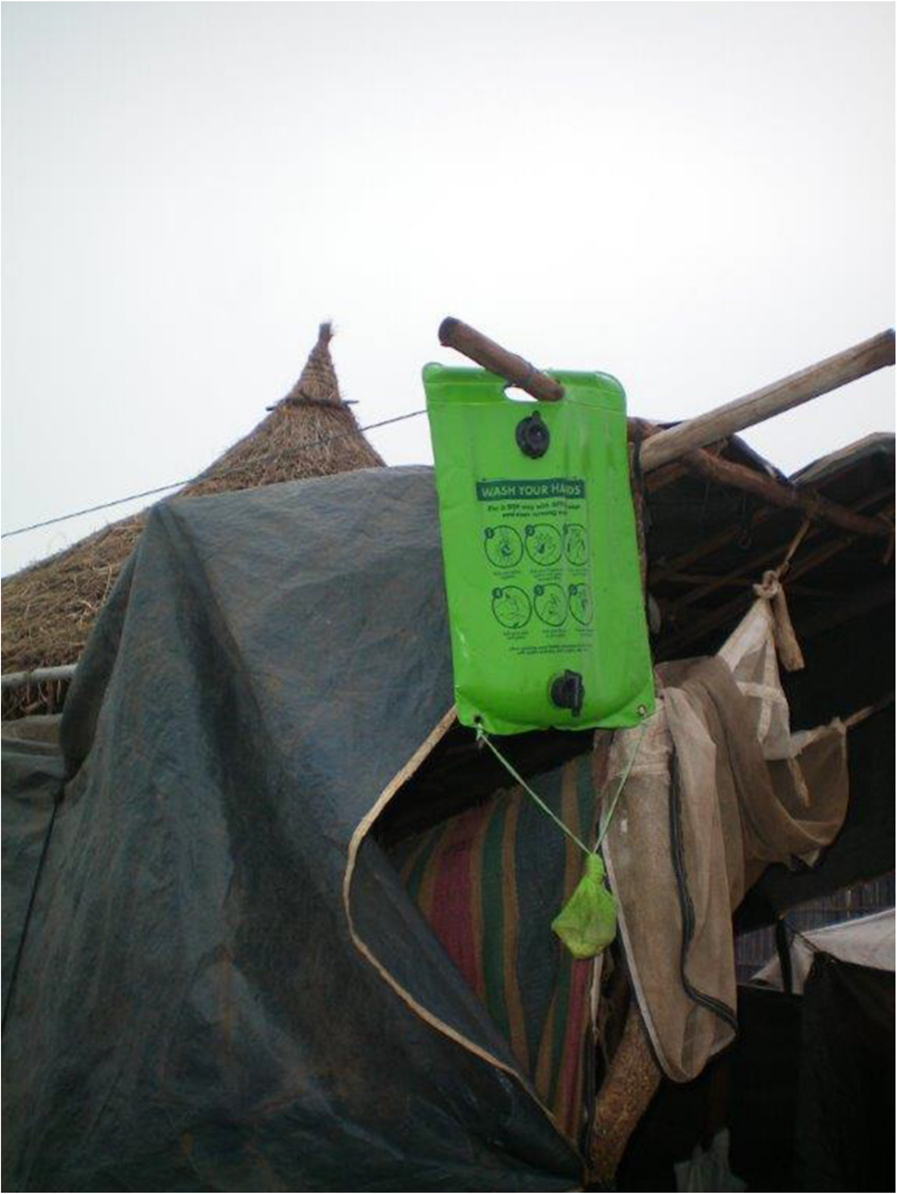
Hand washing bag (HWB)

In February 2012, a HWB with pictorial instructions, an attached spigot and mesh pouch with a bar of Dettol® soap (Fig. 1) was distributed to every household in the Adamazin Transit Center to evaluate the acceptability, durability and use of the bag. The evaluation was composed of four components: 1) focus group discussions (FGDs) at the start and end of the evaluation to obtain in-depth understanding of the HWB; 2) baseline population-based survey to assess the overall WASH situation; 3) three monthly monitoring visits (MVs); and 4) endline population-based survey (Table 1).

**Table 1:** Data collection method, sample size, objectives and location for each component of the study

Activity	Calculated sample size (Actual response)	Objective	Location
Baseline Survey	228 (211)	Assess overall WASH situation in Transit Center	Adamzin
Monitoring Visit	204 (196,203,203)	Follow selected households over three months to monitor HWB use, acceptability and durability	Adamzin
Endline Survey	244 (222)	Assess HWB after six months	Bambasi
Baseline FGD	29 Northern Sudanese	Obtain hand hygiene knowledge and practices; introduce HWB; and formulate questionnaires	Adamazin
	22 Southern Sudanese		
Endline FGD	33 Northern Sudanese	Obtain in-depth information on HWB use, acceptability, and durability	Bambasi

### Setting

We selected Adamazin Transit Center based on four criteria: 1) population size (≤5000 persons); 2) population stability; 3) safety; and 4) accessibility. Due to unforeseen circumstances such as growing insecurity and the increasing number of refugees from South Sudan, Adamazin Transit Center was closed four months after the start of the study and all residents were transferred to Bambasi Refugee Camp, approximately 100 km from the border. Consequently, the endline survey was delayed by two months and took place in Bambasi Refugee Camp, approximately six months post-distribution. The target audience was newly arrived northern and southern Sudanese refugees (arrival date less than four months from start of project). Household members who did not provide consent, aged <16 years, unable to speak Arabic, Amharic, or English, or mentally unable to complete the interview were not eligible to participate.

### Focus group discussions

We conducted six FGDs at baseline (prior to the HWB distribution) to a) identify current hand washing and hygiene knowledge, practices and barriers, b) introduce the HWB, and c) formulate appropriate questions for the baseline and monitoring visit surveys. The baseline FGDs were composed of: a) males with children under-five years of age (7 Northern, 10 Southern), b) females with children under-five years (9 Northern, 12 Southern), c) males aged ≥35 years without children under-five years (5 Northern), and d) females aged ≥35 years without children under-five years (8 Northern). Male and female participants did not come from the same households. Separate FGD sessions were conducted for northern and southern Sudanese for groups a and b only. There were no southern Sudanese refugees living in Bambasi Refugee Camp; therefore, only four FGDs were conducted during the endline evaluation composed of a) 6 males with children under-five years of age, b) 11 females with children under-five years, c) 7 males aged ≥35 years without children under-five years, and d) 9 females aged ≥35 years without children under-five years (Table 1). The endline FGDs were conducted to gather detailed information on the use and acceptability of the HWB. Participants were chosen by community liaisons. A facilitator, translator, and note taker conducted each FGD using a standardized guide [9, 10]. The focus group questions were formulated to address factors in the RANAS model in order to determine possible behavioral determinants that may encourage or hinder the use of hand washing bags.

### Baseline survey

In December 2011, we conducted a baseline survey in Adamazin Transit Center to gather general information on WASH indicators within the transit center two months prior to the HWB distribution. This included questions on water availability, water consumption, hand washing knowledge, hygiene practices, and sanitation. Respondents were adult female heads of households or, if unavailable, adult male heads of households.

#### Data collection

We enumerated and selected households in the Transit Center using stratified systematic random sampling (14 households × 12 blocks, 12 households × 5 blocks) based on new arrivals unaccounted for during our initial estimation. Four teams of two enumerators each (one male, one female) were trained and participated in a pilot prior to the start of the survey. Questionnaires were written in Amharic (Ethiopian national language) and administered in Arabic (Sudanese national language). All technical terminology was standardized during the training. One supervisor was responsible for household selection and data quality control.

#### Sampling frame and sample size

The sampling frame was based on IRC compiled population data. The sample size was calculated assuming proportion of key WASH indicators based on SPHERE standards was 50 % with a precision of +/- 7 %, 95 % confidence limit and a 5 % non-response rate for a total of 200 households. The sample size was adjusted to 228 to account for additional blocks housing new arrivals (see ‘Data collection’ below). This sample was calculated using a formula for finite populations.

### HWB distribution and monitoring visits

IRC distributed one HWB and one bar of Dettol soap to every household in the camp (*n* = 874 HWBs) between Feb 21–25, 2012 with a follow-up visit one week post-distribution to ensure integrity and proper use of the bag. Eighteen bags were non-functional during the post-distribution visit; these households were not included in the sampling frame for the monitoring visits.

#### Data collection

We conducted monthly monitoring visits (MV) at week 4 (MV1), week 8 (MV2), and week 12 (MV3) following the initial distribution. Eight Environmental Health Agents (EHAs), responsible for providing health and hygiene education to assigned households, were trained to visit the *same* preselected households every month for three months to assess the HWBs presence, condition, and use. At every monitoring visit, non-functional bags^2^ were removed from the household and replaced with a new bag. Missing bags were not replaced. One week prior to each monitoring visit, EHAs received a refresher training using the Monitoring Visit Training Guide developed by CDC. Missing and refusing households were not substituted. Four team supervisors were responsible for overseeing the monitoring visits.

#### Sampling frame and sample selection

The sampling frame for the monitoring visits consisted of households with a functioning HWB. The sample size was calculated assuming 80 % of households would be using the HWB with a precision of +/- 5.5 % and a 2 % non-response rate for a total of 200 households. Twelve households per block for a total of 204 households (12 households × 17 blocks) were randomly preselected for the monitoring visits. Tents of selected households were marked with a star and/or note card.^3^

### Endline survey (Bambasi Refugee Camp)

#### Data collection

Households were selected using simple random sample from the list of 367 households (see Sampling frame and sample size section). Eight enumerators were trained following similar methodology as the baseline survey. Data on HWB use, hygiene, hand washing and sanitation practices were collected using a standardized questionnaire.

#### Sampling frame and sample size

Bambasi Refugee Camp was a mix of households from Adamazin Transit Center (i.e. those who had received a HWB) and new arrivals (those who had not a received a HWB). Of the original 856 households who had received a functional bag in the Transit Center, only 367 had moved to Bambasi. The sampling frame for the endline survey was based on this list of 367 households (average household size was 7 persons). The sample size was calculated assuming 50 % of the households were using the HWB with a precision of +/- 7 % and a 25 % non-response rate for a total of 244 households per camp. This sample was calculated using a formula for finite populations.

### Data entry and analysis

Survey data were entered into Epi Info version 7 (CDC, Atlanta, GA) [11]. Data were cleaned and analyzed using SPSS (version 20, IBM Corp., Armonk, NY) [12] in Atlanta. Baseline and monitoring data were weighted during analysis due to the stratified sampling. FGD data were analyzed using the Risk, Attitudes, Norms, Abilities, and Self-Regulatory (RANAS) model [13, 14]. FGD data were organized by the RANAS themes below:

- *Risks*: Hand washing knowledge, perceived vulnerabilities, severity of the situation
- *Attitudes*: Self perceptions and emotions about hand washing with soap, provision of soap and willingness to pay, perception towards HWB
- *Norms*: Cultural beliefs about hand washing, community’s perceptions
- *Abilities*: Ease of use and availability of hardware and software, ability to act on available knowledge and what is needed to maintain their actions
- *Self-regulatory:* specific use, self-effective vs. non self-effective approach, coping mechanism, issues related to planning

The primary (but not limited to) proxy indicators used to ascertain acceptability and use were:

- % of hanging bags at the time of visit (baseline, MVs, endline)
- % of households having bags with water at the time of visit (baseline, MVs, endline)
- % of respondents reported using the HWB as their primary hand washing device (endline)
- % of respondents reported using the HWB last time they washed their hands (endline)

To assess durability of the bag, a survival analysis using the Kaplan-Meier estimator was performed based on the time to failure of each bag (i.e. break, leak, tear, etc.); HWBs that did not fail were considered to survive. Only monitoring visit data (three months) were used for the survival analysis.

All surveys were maintained under lock and key.

### Ethical considerations

No identifying information was maintained and verbal informed consent was obtained prior to administration of the questionnaires. The study was approved as non-human subjects research under category IC (program evaluation) based on CDC Policy on Defining Public Health Research and Non-research.

## Results

### Background

The baseline survey had 211 respondents (17 absent/refusals) of which 86.9 % were female. The endline survey, conducted six months post-distribution, included 222 households^4^ (2 refusals) of which 84.6 % were female respondents. Response rates were comparable for the three monitoring visits (81.5 %–88.8 %). The mean length of stay in Adamazin was 66 days at the time of the baseline survey and 80 days in Bambasi. Mean household size was five during both baseline and endline surveys (Table 2).

**Table 2:** Household demographics during baseline and endline surveys

Demographics	Baseline	Endline
	*N* = 211	*N* = 222^a^
Mean household size (range)	5.1 (1–25)	4.9 (1–11)
Total household members	1094	1088
Total males (mean)	88 (4.0)	163 (4.8)
Total females (mean)	1006 (5.3)	921 (4.9)
Female to male ratio	8.7	17.7
Number of Children U5 (%)	273 (25)	321 (29.5)
Mean number of children U5 per hh	1.3	1.4
Mean number of days in camp (range)	66.0 (0–155)	79.9 (0–120)

^a^Missing sex for 1 respondent

### WASH environment and knowledge at baseline

In Adamazin, all respondents (100 %) obtained water from a nearby tap stand and reported mean collection time was 15 min. The average amount of water collected was 10 liters per person per day (range 0–57), less than UNHCR standards of 20 l/p/d, [15] and varied by household size. Households with fewer members collected larger volumes per person than larger households. The mean number of water storage containers per household was two. Nine households (5.0 %) did not have any containers.

Almost 50 % of households interviewed did not have soap on the day of the baseline survey. Among households with soap, less than 30 % reported using it for hand washing (data not shown). Although monthly soap distribution was conducted in Adamazin Transit Center during the three months of the monitoring visits, the presence of soap specifically for hand washing (presence of soap attached to or near the HWB) declined over time; from 63.2 % (CI: 55.2–70.6) during MV1 to 46.0 % (CI: 37.9–54.4) during MV3. Availability of any soap also declined; from 31.9 % of households having no soap (CI: 24.8–40.0) during MV1 to 51.6 % (CI: 43.2–60.0) during MV3. Presence of hand soap was also low in Bambasi Refugee Camp; only 8.2 % of households had soap attached to or near the HWB upon observation at the end of the six month study period (Table 3).

**Table 3:** Soap availability over time

	MV1		MV2		MV3		Endline	
	*N* = 158		*N* = 150		*N* = 141		*N* = 222, 209^a^	
	Number	% (95 CI)	Number	% (95 CI)	Number	% (95 CI)	Number	% (95 CI)
Soap present with HWB	101	63.2 (55.2–70.6)	72	48.0 (39.8–56.4)	64	46.0 (37.9–54.4)	17	8.2 (5.2–12.7)
No soap in HH	49	31.9 (24.8–40.0)	45	30.2 (23.3–38.3)	74	51.6 (43.2–60.0)	9	4.1 (2.1–7.6)

^a^These were two separate questions for the endline survey, therefore *N* = 222 for ‘Soap present with HWB’ and *N* = 209 for “No soap in HH’

 Among baseline FGD participants, h and washing knowledge for critical times was high, however key barriers were insufficient soap supply, lack of “al-brik” (2 L water ewer), lack of hand washing stations near the latrines and the unpleasant smell of the distributed soap which could be considered motivators for hand washing and use of the bag. There was a strong self-regulatory attitude among the group in terms of hand washing in general. Initial observations towards the HWB were positive. Although participants could not read the English instructions printed on the bag, they felt the pictures were easy to understand and follow.

### Use

**Fig. 2:**
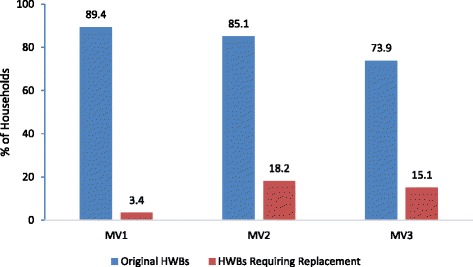
Households with original HWBs and those requiring replacement during three-month period

**Fig. 3:**
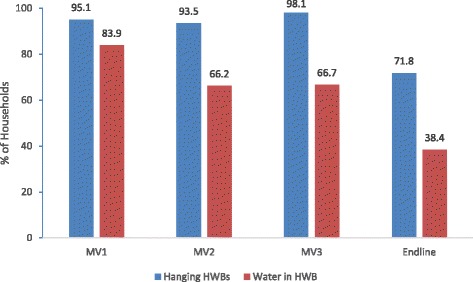
Households with hanging HWBs and water in HWBs during the study period (Note: MV households were not the same as Endline households)

Over three months of the monitoring visits, ownership of an original HWB fell from 89.4 % to 73.9 % (Fig. 2). Almost all of the bags were hanging during the three monitoring visits (MV1: 95.1 %, MV 2: 93.5 %, MV3: 98.1 %), however the percent of bags with water fell from 83.9 % to 66.7 %. After completion of the study (endline), 92.8 % of *newly sampled households* had an original HWB, 71.8 % of observed bags were hanging, but only 38.4 % of all HWBs had water at the time of visit (Fig. 3). Ninety-one percent of respondents reported the primary purpose of the bag was for hand washing, but less than one-half (45.9 %) used the HWB as their primary hand washing device and only one-third (36.4 %) reported using the HWB last time they washed their hands (Table 4). Approximately one-third (31.1 %) of respondents reported that no one in their household uses the HWB. Some reasons included inability to hang the bag, the bag was defective, or there was no soap. Households with water in the bag were more likely to use the HWB as the primary device to wash their hands (p < 0.0001), use the HWB last time they washed their hands (*p* < 0.0001), and refill the HWB daily (p < 0.02) compared to households without water in the bag; availability of soap in the household and hand hygiene knowledge were not significantly different.

**Table 4:** Primary device used for handwashing and device used for previous hand washing event at endline survey

Vessel	Primary device for hand washing *N* = 209		Device used last time for hand washing *N* = 209	
	Number	% (95 % CI)	Number	% (95 % CI)
Another container	111	53.1 (46.3–59.8)	130	62.2 (55.4–68.6)
Hand washing bag	96	45.9 (39.2–52.8)	76	36.4 (30.1–43.2)
Hand washing station- latrine	1	0.5 (0.1–3.4)	3	1.4 (0.5–4.4)
Other	1	0.5 (0.1–3.4)	0	0

Most endline FGD participants felt the quantity of soap in the camp was insufficient which may have hindered the use of the HWB. A common complaint was lack of promotion on use of the bag in Bambasi compared with Adamazin. One respondent stated, “In Adamazin, the bag was filled with water every day because there was a supervisor to oversee it and reminding us of the importance, but here [Bambasi], nobody asks the people about it.”

### Acceptability

Almost all of the households at endline reported liking the bag (99 %). Of those households who reported using the bag (61.2 %), 77.1 % said the pictorial instructions were clear, and 56.2 % felt the bag was too small (Table 5). Endline FGD participants liked that the bag was stationary (‘no one can move it’ or ‘children cannot play with it’) while others preferred the 2 L al-brik because it was easier to use (to carry to the latrine and/or not as heavy). Participants also stated they liked the attached mesh soap bag and clear instructional pictures. Some FGD participants felt the bag was not strong enough or had a bad smell if left in the sun too long.

**Table 5:** Acceptability of HWB at endline survey

	Number	95 % (CI)
Did you like the HWB, overall? (*N* = 207)^a^		
Yes	205	99.0 (96.2–99.8)
No	2	1.0 (0.2–3.8)
HWB size (*N* = 144)^a^		
Acceptable	53	36.8 (29.2–45.1)
Too small	81	56.2 (48.0–64.2)
Too big/heavy	8	5.6 (2.8–10.8)
Don’t know	2	1.4 (0.3–5.5)

^a^Excludes HHs that reported ‘no one’ uses HWB (65 HHs)

### Durability

**Fig. 4:**
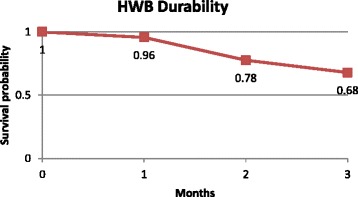
HWB Durability

A total of 6 (3.4 %), 27 (18.2 %), and 21 (15.1 %) bags were replaced during MV1, MV2, and MV3, respectively, because they were not functional (broken, leaking, etc.). The survival analysis revealed a 68 % probability that a HWB would be functional or “survive” for at least three months in an emergency setting. In other words, approximately 2/3 of households should have a functional HWB at the end of three months. Mean survival time of the HWB was 2.73 months (Fig. 4). Endline data were not included in the analysis. It was also not possible to extrapolate data for longer time period due to insufficient information about the physical properties of the HWB.

## Discussion

As per the baseline findings, access to water and sanitation were below minimum standards in Adamazin, with the mean volume of collected water estimated to be approximately 10 l/p/d which could inhibit an enabling environment for proper hand washing practices. Overall, the HWB was well received according to FGD participants. Despite concerns regarding the durability of the plastic in hot climates, participants liked the overall physical properties of the bag and felt the device would be useful in encouraging hand washing. The majority of households hung the HWB and had water in the bag during the monitoring visits indicating use. The bag itself seemed durable over the three month period considering mean survival rate was 2.73 months. However, long-term outcomes (beyond three months) in an emergency setting are unclear. The majority of households sampled at the endline had retained their original HWB and an overwhelming number reported liking the HWB, proxy indicators such as water in the bag or a hanging bag to gauge use revealed variability. For example, while 72 % of households had a bag hanging during the endline survey, only 39.2 % of all households had water in the bag. Additionally, the majority of endline respondents used another container to wash their hands and only one-third of households reported using the HWB the last time they washed their hands. The low levels of reported use measured by these proxy indicators at six months indicates decreasing acceptability over time or a reflection of potential differences between Adamazin Transit Center and Bambasi Refugee Camp. For example, 46.0 % of households had soap with the bag (hand washing soap) during the monitoring visits in the Transit Center while only 8.2 % had hand soap at the endline in Bambasi. Although the majority of households had soap present at the time of endline survey; this soap was not attached to or near the HWB, and therefore, may have been used for multiple purposes other than hand washing. Similarly, the EHAs in the Transit Center provided repeated reminders to use the bag as well as additional health and hygiene information during the monitoring visits, which may have influenced behavior. Concerns regarding bag durability remained high among the endline FGD participants, reiterating issues raised during the baseline discussions. A stronger, heat-resistant bag that would not easily breakdown, or change the smell of the water may also increase acceptance of the HWB.

### Limitations

There were several limitations in this study. First, there was loss to follow-up of households who did not move from Adamazin to Bambasi. There may have been potential differences between the households that did not move to the permanent camp (they remained within the host community or returned to Sudan) compared with those households that did move (i.e., education level, employment ability, socioeconomic status, etc.). In addition, the move to Bambasi may have resulted in a loss of the HWB itself. Differences between the two settings may have contributed to differences in our findings. Second, it is possible that observations and reporting were affected by the Hawthorne effect; households selected for the repeat monitoring visits were aware of the monthly checks, and therefore, modified their behavior (hanging or filling the HWB) in preparation for the visits, which were scheduled around the same time each month. Third, study tools were written in Amharic, but translated into Arabic during the interviews. This on-the-spot translation may have resulted in improper or inaccurate interpretations of the questions. All efforts were made during the training to reduce this. Lastly, although this evaluation assessed soap availability at the household level, we did not assess whether households received the full ration of 450 g/person/day or if the amount received was sufficient for their needs. If insufficient, they may have prioritized the soap they did have for purposes other than hand washing or use of the HWB.

## Conclusions

This was the first known acceptability study of a portable hand washing device at the household level during an acute emergency among a recently displaced population. In this evaluation, the HWB performed well during the early phases of the emergency (first three months) when the risk of disease transmission is usually highest and basic water, sanitation and hygiene services are often insufficient; however, longer term outcomes are unclear. It is also not known whether the HWB influenced hand washing behavior among the camp population, as assessing hand washing behavior in a very short time is challenging in these environments. There is no independent verification method to evaluate hand washing behavior as both recall and direct observation are often limited by the potential biases introduced [16]. Distributing the bag to households, as part of a household arrival package, may initiate early hand washing behavior and serve as a stopgap measure until longer term provisions are made. Regular soap distribution and increased hygiene promotion along with improvements to the bag (strength, size) may also help increase hand washing behavior. The HWB has the potential to be a good option for a hand washing intervention during the early phases of an emergency; however additional WASH activities such as adequate water, adequate hand soap provision and messaging must coincide for an enabling environment.

### Ethics approval

This study was exempted from review by the Institutional Review Board of the CDC as the primary intent of the study was determined to be non-research.

## Abbreviations

ARRA: The Administration for Refugee and Returnee Affairs
BGRS: Benishangul-Gumuz Regional State
CDC: Centers for Disease Control and Prevention
EHAs: Environmental Health Agents
FGDs: Focus group discussions
HWB: Hand washing bag
IRC: The International Rescue Committee
MV: Monitoring visits
RANAS: Risk, Attitudes, Norms, Abilities, and Self-Regulatory
SPLA-N: South Sudan People’s Liberation Army
UNHCR: UN High Commissioner for Refugees
WASH: Water, sanitation, and hygiene

## Footnotes

1 Cost per bag is USD 4.13.

2 A bag was considered non-functional if it was broken, leaking, torn, missing mesh bag for soap, missing/broken cover or any other problem that prevents the use of the bag in its entirety.

3 IRC obtained permission from the Administration of Refugees and Returnees Administration (ARRA) in Assosa, to mark the tents.

4 This was a new sample of randomly selected households (not same preselected households interviewed for the monitoring visits).
